# Comparison of the preconditioning effect of different exercise training modalities on myocardial ischemia-reperfusion injury

**DOI:** 10.1371/journal.pone.0295169

**Published:** 2023-12-05

**Authors:** Reihaneh Mohammadkhani, Kamal Ranjbar, Iraj Salehi, Alireza Komaki, Ebrahim Zarrinkalam, Parsa Amiri

**Affiliations:** 1 Neurophysiology Research Center, Hamadan University of Medical Sciences, Hamadan, Iran; 2 Department of Physical Education and Sport Science, Bandar Abbas Branch, Islamic Azad University, Bandar Abbas, Iran; 3 Faculty of Physical Education and Sport Sciences, Department of Physical Education, Islamic Azad University, Hamedan Branch, Hamedan, Iran; Max Delbruck Centrum fur Molekulare Medizin Berlin Buch, GERMANY

## Abstract

The study of exercise preconditioning can develop strategies to prevent cardiovascular diseases and outline the efficient exercise model. However, the exercise type with the most protective effect against ischemia-reperfusion injury is unknown. In this study, we examined the effects of three kinds of exercise preconditioning on myocardial ischemia-reperfusion in adult rats and explored the possible underlying mechanisms. Male Wistar rats subjected to ten weeks of endurance, resistance, and concurrent training underwent ischemia (30 min) and reperfusion (120 min) induction. Then, infarction size, serum levels of the CK-MB, the redox status, and angiogenesis proteins (VEGF, ANGP-1, and ANGP-2) were measured in the cardiac tissue. Results showed that different exercise training modes have the same reduction effects on infarction size, but ischemia-reperfusion-induced CK-MB was lower in response to endurance training and concurrent training. Furthermore, cardiac VEGF levels increased in all three kinds of exercise preconditioning but ischemia-reperfusion-induced ANGP-1 elevated more in endurance training. The cardiac GPX activity was improved significantly through the resistance and concurrent exercise compared to the endurance exercise. In addition, all three exercise preconditioning models decreased MPO levels, and ischemia reperfusion-induced MDA was lower in endurance and resistance training. Overall, these results indicated that cardioprotection of exercise training against ischemia-reperfusion injury depends on the exercise modality. Cardioprotective effects of aerobic, resistance, and concurrent exercises are due to different mechanisms. The preconditioning effects of endurance training are mediated mainly by pervasive angiogenic responses and resistance training through oxidative stress amelioration. The preconditioning effects of concurrent training rely on both angiogenesis and oxidative stress amelioration.

## Introduction

Myocardial ischemia-reperfusion injury is the most important cause of mortality in the world, and more than 20 percent of mortality in the US is caused by Myocardial ischemia-reperfusion [1]. Theoretically, reperfusion may ameliorate cardiac function; however, it has been confirmed that reperfusion leads to more injury in the myocardium due to oxidative stress and inflammation [2–4].

Ischemic preconditioning is short episodes of ischemia-reperfusion, making the myocardium resistant to prolonged coronary artery occlusion and decreasing following infarct size [5]. Different physiological and pathophysiological mechanisms, such as inflammation, apoptosis, oxidative stress, and angiogenesis, are activated or inactivated in response to ischemic preconditioning, determining the severity of damage caused by ischemia. Today, the preconditioning effect of exercise on ischemia-reperfusion injury is well-established [6,7]. The capacity of acute exercise to activate several pathways protecting the heart from possible ischemic damage is defined as exercise preconditioning. Rodent-related studies revealed significant cardioprotective effects of resistance training and aerobic exercise against infarction [8–11]. Concerning the data, repeated bouts of endurance exercise can modify the expression of several mitochondrial proteins and cardioprotective mediators [12].

Training specificity is critically important since it affects the subsequent adaptations of the tissue [1,13,14]. The adaptations created by resistance and endurance training have conflicting outcomes [15]. Resistance exercise resembles remote ischemic conditioning [16]. It increases muscle force, glycolytic activity, and intramuscular ATP/phosphocreatine reserves with close to no change in aerobic capacity. It can also result in fiber hypertrophy and reduce the mitochondrial and capillary density of the muscle [13,15,17,18]. Endurance training has been shown to improve aerobic capacity and elevate mitochondrial and capillary density [13,15,17]. Studies have shown conflicting results concerning the effects of resistance and endurance training methods performed together (concurrent training) [19]. The reason behind these contradictory outcomes may be due to diverse methodologies.

It has been shown that ischemia-reperfusion injury is associated with ROS production [7,20]. The adverse effects induced by ROS generation include alteration in intracellular pathways and redox signaling, cellular dysfunction, mitochondrial DNA (mtDNA) damage, extracellular matrix remodeling, apoptosis, and contractile function impairment [21]. More importantly, regular physical activity increases antioxidant activity and decreases lipid peroxidation [22].

Angiogenesis is an effective process of post-ischemic restoration that helps supply oxygen and nutrition to the infarcted tissue, preventing heart failure [23]. Growth factors can stimulate an angiogenic response, altering the endothelial cells and vessel formation from preexisting capillaries [24]. The angiopoietin family, consisting of angiopoietin-1 (ANGP-1) and angiopoietin-2 (ANGP-2), are crucial growth factors for new blood vessels to sprout. These factors modify VEGF function to create vascular remodeling and maturation. ANGP-2 causes Endothelial instability, paving the way for endothelial activation by VEGF-A. On the other hand, ANGP-1 serves as a supportive factor for new vessels [25–28] and prevents endothelial death [29]. Physical exercise has been shown to modify the expression of angiopoietin and VEGF-A and change their protein levels [30].

In contrast to endurance or resistance exercise, few studies investigated the effects of concurrent training on myocardial ischemic injury. However, there are still significant gaps in the evidence indicating what types of training might be most effective at reducing ischemia-reperfusion injury. Furthermore, Identifying underlying physiological mechanisms involved in reducing ischemia-reperfusion injury can help with the treatment. Regarding the paucity of data and results from different models of experimental designs, this study aims to evaluate the ameliorative effects of exercise preconditioning through three different training modes on myocardial ischemia-reperfusion injury and explore the probable underlying mechanisms.

## 2. Material and methods

### 2.1. Animals

Fifty male Wistar rats, weighing 200–250 g, were used and kept under the standard conditions (22±2°C temperature and 12-h light-dark cycle) with ad libitum access to water and rodent chow. The animal treatments were performed strictly under the National Institutes of Health Guide for the Care and Use of Laboratory Animals, with approved experimental protocol.

### 2.2. The experimental design

The animals were randomly divided into five groups (N = 10/group), including the sedentary sham group, the INF (infarcted) control group, INF+ EE (infarcted rats with endurance exercise intervention), INF+ RE (infarcted rats with resistance exercise intervention), and the INF+ CE (infarcted rats with concurrent exercise intervention). After the grouping, rats underwent ten weeks of exercise training before the ischemic induction. Then, serum measurements, tissue assessments, and redox status determination were performed.

### 2.3. Training interventions

The exercise intervention consisted of training sessions before the ischemia-reperfusion injury. The training volume was equal in the three exercise groups.

#### 2.3.1. Endurance training

The rats were trained five days a week for ten consecutive weeks. The overload principle was used to determine the intensity of training. The first week’s routine consisted of running on a treadmill at a speed of 17 meters per minute for 10 minutes. The running time was increased by 10 minutes to 60 minutes per session between the second and the sixth weeks. The rats underwent exercise sessions for 60 minutes on a treadmill at a speed of 30 m/min, approximately equivalent to 90% VO_2_ max, during which the treadmill was tilted 10 degrees in the seventh to tenth weeks [31].

#### 2.3.2 Resistance training

To familiarize the animals with the device and reduce their stress, they were placed on a 36-step ladder at 85° for three sets with two bouts without weight for three days. Resting periods were three minutes between each set and fifteen seconds between repetitions. The standard exercise routine was followed for ten weeks, five times a week, and each day consisted of three sets with four bouts. Repetition was defined as one climb of the ladder, and the set was defined as completing four consecutive repetitions. Resting periods between each set and repetitions in the primary training protocol were similar to familiarize time. A cloth bag containing weights was attached to the base of the tail with tape. In the first three weeks, the animals trained with 20, 40, and 60 percent of their body weight. In the next three weeks, the animals trained with 80, 100, and 120 percent of body weight. In the last four weeks, the animals trained with 140, 160, and 180 percent of their body weight [31].

#### 2.3.3 Concurrent training

Ten weeks of concurrent exercise were performed in parallel with aerobic and resistance exercises five days a week. Half the time of each session was allocated to resistance training and the other half to endurance training, alternately changing order [31]. In this group, exercise intensity progressed in both training modalities at the same rate as in rats performing only one type of exercise.

### 2.4. Ischemia-reperfusion operation

Seventy-two hours after the last exercise session, rats underwent an ischemia-reperfusion operation. Transient occlusion of the left coronary artery was used to induce ischemia-reperfusion injury. The rats, anesthetized by the intraperitoneal (IP) injection of sodium pentobarbital (60 mg/kg body weight, IP.), were injected with Heparin (1000 IU/kg; IP) to prevent coagulation. Soon after, respiration was stabilized using a rodent respiratory apparatus (Small Animal Ventilator, Model 683, Harvard Apparatus) with the air of the room (15 ml/kg stroke volume and 60–70 Breaths/min). The chest was cut open, and the pericardium was excised to expose the coronary artery. A 6–0 silk suture was wrapped around the left coronary artery, with its ends enclosed in a polyethylene tube to form a snare. The tightened suture ligated the coronary artery, inducing ischemia injury for 30 minutes. Then, the ischemic myocardium underwent 120 minutes of reperfusion [32]. The given time is chosen based on the previous studies [8,16]. Body temperature was maintained at normal levels via a heating pad. At the end of the experiment, the left ventricle was dissected for further analysis. One rat from each group died during exercise training or an ischemia-reperfusion operation. Out of nine rats in each group, four were used for histological evaluation to measure the area at risk and infarction size, and the other five rats were used to harvest fresh tissue (stored in liquid nitrogen) for biochemical analysis to assess angiogenic and oxidative stress indices.

### 2.5. Assessment of myocardial ischemia-reperfusion injury

The severity of the damage caused by myocardial ischemia-reperfusion, the size of the infarction, and creatine kinase myocardial band isoenzyme (CK-MB) were assessed. 2 ml of blood was drawn from the Vena cava in all rats in each group and measured using a CK-MB Assay kit and an autoanalyzer (Roche Hitachi Modular DP Systems, Mannheim, Germany), under the manufacturer’s instruction (Pars Azmoon, Iran) to evaluate CK-MB serum levels. Then, the blood samples were centrifuged at 3000 rpm for 5 min at 4°C. After, the supernatants were stored at −70°C for further investigations.

### 2.6. Measurement of infarct size

After the reperfusion process and the re-tightening of the left coronary artery, 2 ml of 0.02 percent Evans blue solution (Sigma-Aldrich, St Louis, MO, USA) was injected into the femoral artery to distinguish the non-risk area of the heart (blue-stained) from the area at risk (AAR- the area of each slice that did not turn blue in response to perfusion with the Evans blue) in four rats in each group. Then, the heart was cryopreserved immediately at -70°C. 1mm Cross-sections were prepared by the transverse cutting of the samples from base to apex. Images from these cross-sections were captured from both sides using a scanner (HP Scanjet G2410, Flatbed Scanner). Subsequently, the slices were incubated with 1 percent 2,3,5-triphenyl tetrazolium chloride (TTC, Sigma-Aldrich, St Louis, MO, USA) and 0.1M phosphate buffer for 20 minutes at 37°C. The cardiac slices were then preserved in 10% formaldehyde for one day before being scanned again. The total slice area, the region at risk, and the extent of the infarct were determined. The samples underwent another scanning following their fixation in 10% formaldehyde. Infarction size is the portion of the area at risk that did not turn red after TTC staining and remained white. The AAR was expressed as a percentage of the left ventricle (AAR/LV), and the size of the infarct area was measured as the ratio of the white portion to the AAR [20,33].

### 2.7. Assessment of myocardial oxidative stress

Five remaining rats were euthanized with a sodium pentobarbital overdose, with their left ventricular tissues collected to assess angiogenic and oxidative stress indices. Homogenates, prepared at a 1:10 weight-to-volume ratio in a 0.1 mol/L phosphate buffer (pH 7.4), were centrifuged for 10 minutes at 1500 g (4°C). The supernatant was used for myocardial redox evaluation. Following the Paglia method, glutathione peroxidase (GPx) activity (U/mg protein) in ventricular heart tissue was determined using a GPx assay kit (Randox Life Sciences, Crumlin, UK) under the manufacturer’s protocol. Then, a Hitachi U-2000 spectrophotometer (Tokyo, Japan) was used to measure any decrease in absorbance at 340 nm [34]. The GSH content (U/mg protein) was measured according to Ellman (1959) and assayed spectrophotometrically at 412 nm [35]. The Aebi method (1984) was used to determine the catalase (CAT) activity (U/mg protein) by a UV-spectrophotometer at 240 nm, through which a decrease in absorbance indicates the degradation of hydrogen peroxidase to oxygen and water [36]. MDA levels of the heart tissue (nmol/mg protein) were measured following the Niehaus method (1968) using a UV-spectrophotometer at 532 nm to read the absorbance of the colored layer [37]. According to Huang et al., MPO activity was measured at 37°C on a spectrophotometer at 655 nm for three minutes and expressed as nmol/mg protein [38].

### 2.8. Assessment of myocardial proteins

**Fig 1:**
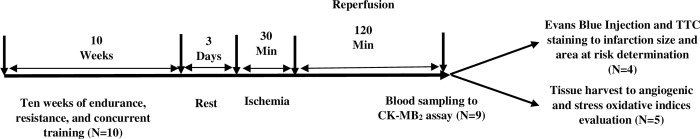
Experimental timeline.

The heart tissue was homogenized with a homogenizer after weighing, grinding, and then lysing using the lysis buffer. A solution consisting of the protease inhibitor and extraction protein, proportional to the homogenized tissue, was made according to the instructions of the kit company (Kiazist Life Sciences, Iran). The cocktail was centrifuged at 20,000 × g for 10 min. Total protein concentration was measured by the BCA method. ANGP-1, ANGP-2, and VEGF were measured using an ELISA kit (Cusabio, USA). The Experimental Timeline is shown in Fig 1.

### 2.9. Statistical analysis

SPSS software (version 23) was used for statistical analyses. The effects of training intervention were studied using the Kruskal-Wallis H test. The U Mann-Whitney tests were used to determine significant differences between groups. The data were presented as the Mean ± standard error of the mean (SEM). P<0.05 values were considered to be significant.

## 3. Result

### 3.1. Infarction size

**Fig 2:**
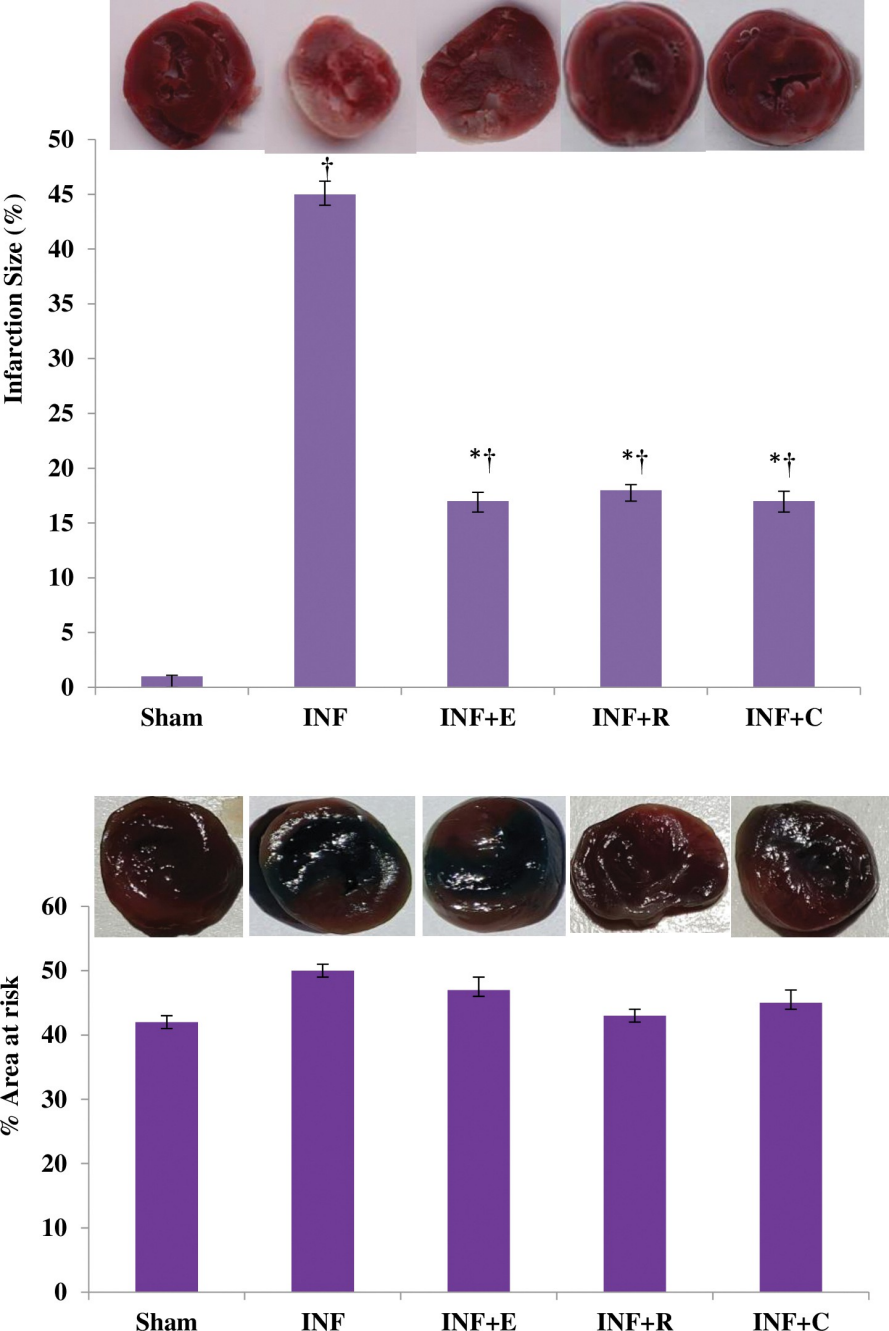
Representative pictures of Area at risk and myocardial infarction size (%) in experimental groups (N = 4/group). * Significant difference vs. INF group, † Significant difference vs. Sham group.

The AAR was not different between experimental groups (χ^2^ = 9.93, p = 0.052). According to the statistical analysis, there was a significant difference in the infarction size among experimental groups (χ^2^ = 14.9, p = 0.005). The infarction size decreased significantly compared to the control group in response to various training methods following the regional ischemia-reperfusion. Noteworthy, myocardial infarct size was similar among training groups, noting that the type of exercise does not affect the reduction magnitude (Fig 2).

### 3.2. CK-MB_2_ serum levels in different exercise preconditioning modes

**Fig 3:**
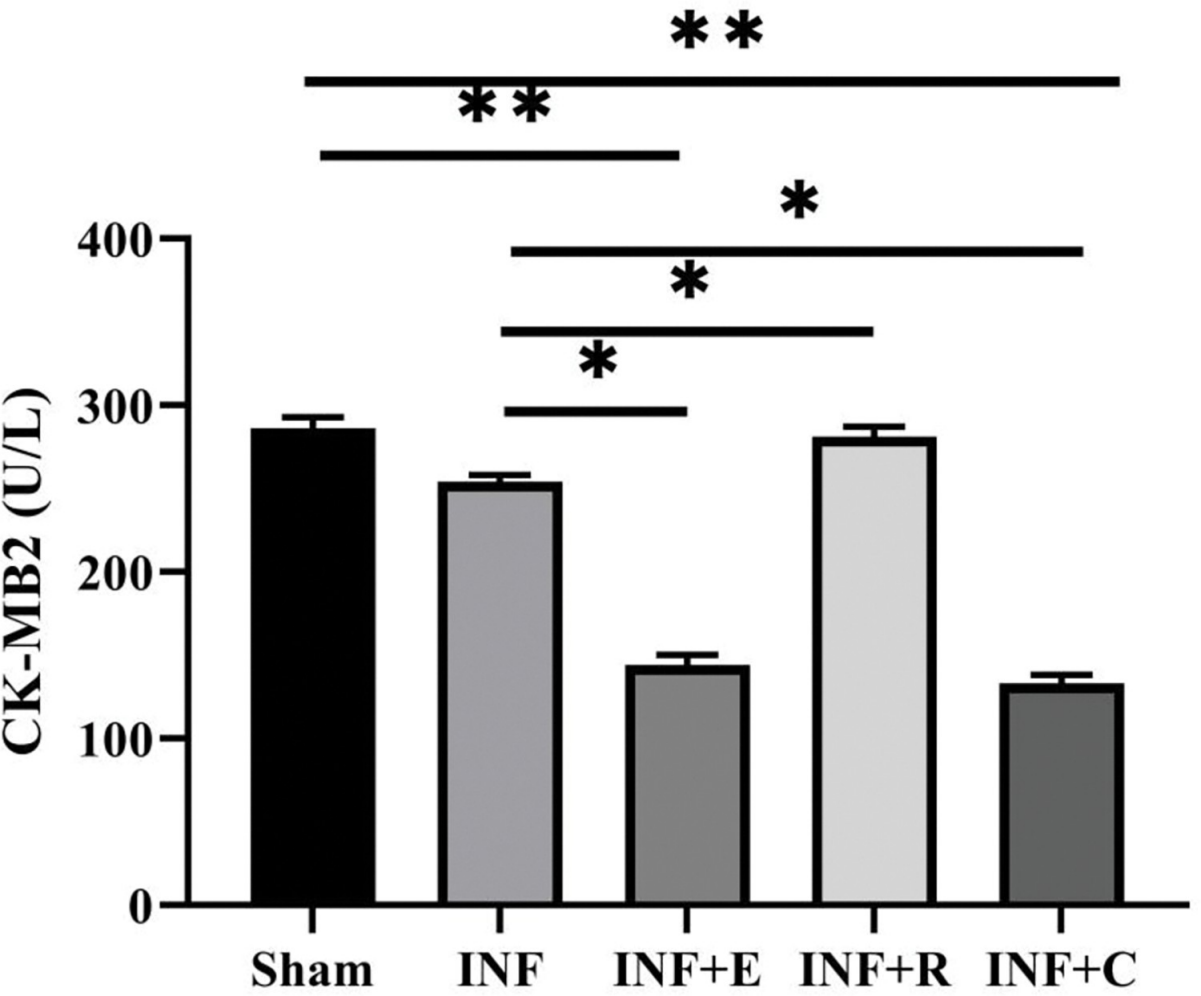
Effect of exercise preconditioning on the serum CK-MB_2_ level. Groups: The sedentary sham group, the INF (infarcted) control group, INF+ E (infarcted rats with endurance exercise intervention), INF+R (infarcted rats with resistance exercise intervention), and the INF+C (infarcted rats with concurrent exercise intervention. * vs. Sham group and ** vs. INF or control, P< 0.05. Bars represent the Means ± SEM (n = 9/group).

As shown in Fig 3, serum CK-MB_2_ differed between experimental groups (χ^2^ = 36.1, p = 0.0001). Multiple comparisons showed that ischemia reperfusion-induced CK-MB_2_ were lower in endurance training (p = 0.001) and concurrent (p = 0.0001) groups compared to the control group, but resistance training elevated serum CK-MB_2_ after ischemia-reperfusion (p = 0.003).

### 3.3. Angiogenic protein levels in different exercise preconditioning modes

**Fig 4:**
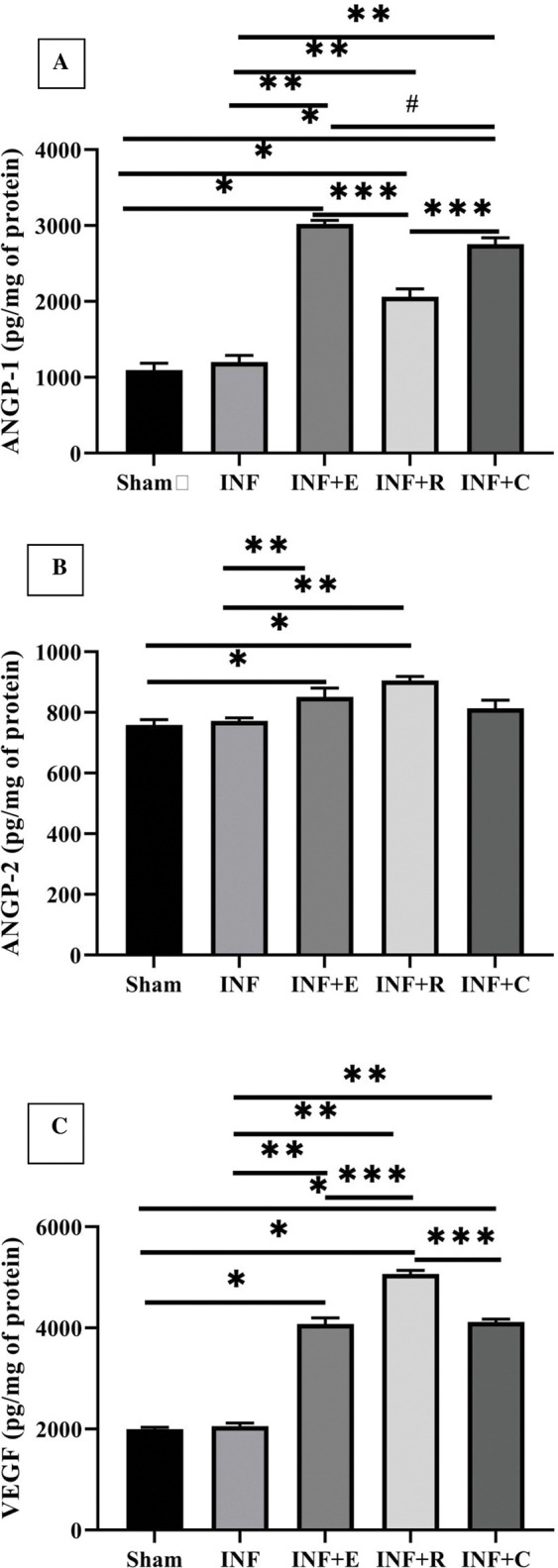
Effect of exercise preconditioning on the angiogenic proteins level. A) The cardiac protein level of ANGP-1. B) The cardiac protein level of ANGP-2. C) The cardiac protein level of VEGF. Groups: The sedentary sham group, the INF (infarcted) control group, INF+ E (infarcted rats with endurance exercise intervention), INF+ R (infarcted rats with resistance exercise intervention), and the INF+ C (infarcted rats with concurrent exercise intervention. * vs. Sham group and ** vs. INF or control, *** vs. INF-R group, **#** vs. INF+E group, P< 0.05. Bars represent the means ± SEM (n = 5/group).

ANGP-2 (χ^2^ = 15, p = 0.005), ANGP-1 (χ^2^ = 21.52, p = 0.0001) and VEGF (χ^2^ = 21.03, p = 0.0001) were significantly different between experimental groups. Cardiac angiogenic protein levels were increased by exercise preconditioning; however, each training mode had different effects and was insignificant in some cases. The statistical analysis showed that endurance, resistance, and concurrent training preconditioning increase the ANGP-1 protein significantly (Fig 4A). ANGP-1 elevation in aerobic training was more than in concurrent and resistance groups (p<0.05). Also, the ANGP-2 results showed a significant increase in aerobic and resistance training groups (Fig 4B). The VEGF protein levels in the heart increased significantly in all three training models (Fig 4C). There was no significant difference between INF and sham groups concerning the studied proteins, but cardiac VEGF in resistance groups was more than in aerobic and concurrent groups (p = 0.009).

### 3.4. Cardiac antioxidant and oxidant in the effects of three kinds of exercise preconditioning

There was a difference in cardiac GPX among groups (χ2 = 20.1, p = 0.0001). Cardiac GPX in INF groups was less than in sham groups (0.009). Statistical analyses showed that different modes of training elevated cardiac GPX. GPX was not different between training groups. Also, no significant effect on GSH (χ^2^ = 2.8, p = 0.5) and Catalase (χ^2^ = 3.4, p = 0.44) activity was observed following exercise preconditioning. Cardiac GSH and catalase were not different between experimental groups.

**Fig 5:**
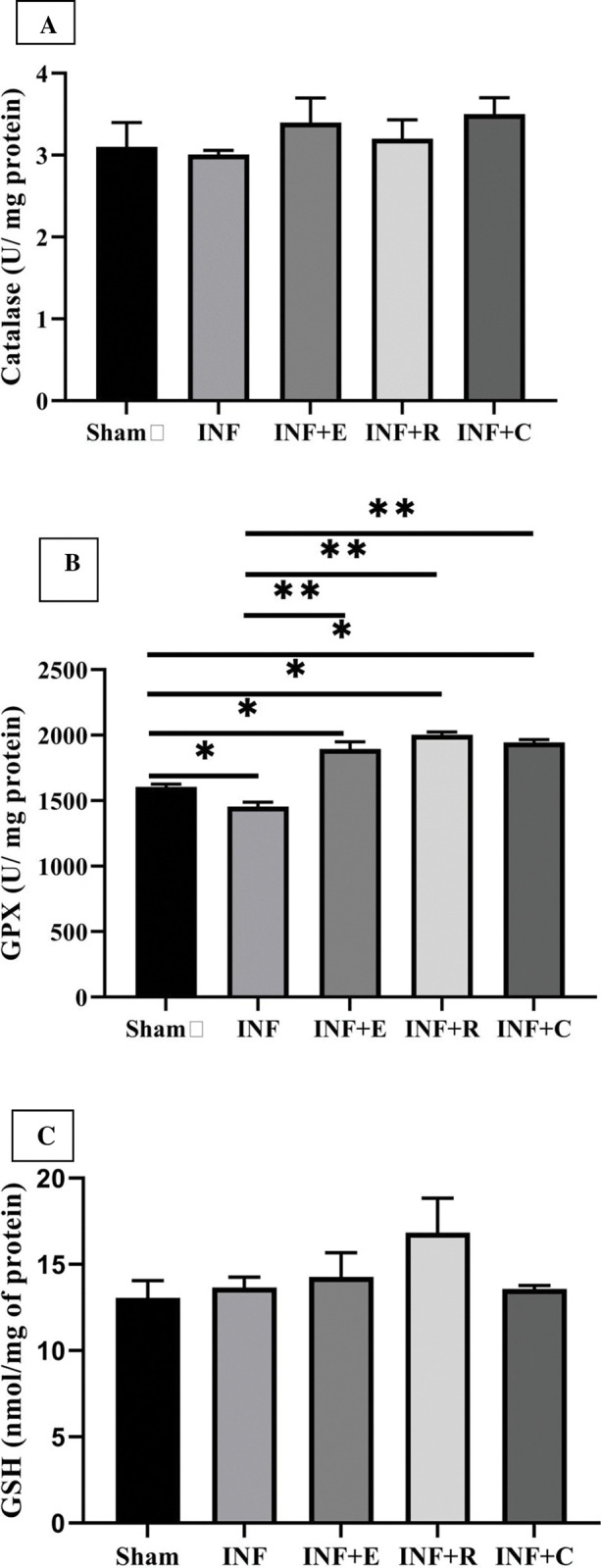
Effect of exercise preconditioning on the cardiac antioxidant level. A) Activity of Catalase. B) Activity of Glutathione peroxidase. C) Activity of Reduced Glutathione. Groups: The sedentary sham group, the INF (infarcted) control group, INF+ E (infarcted rats with endurance exercise intervention), INF+ R (infarcted rats with resistance exercise intervention), and the INF+ C (infarcted rats with concurrent exercise intervention. * vs. Sham group and ** vs. INF or control, P< 0.05. Bars represent the means ± SEM (n = 5/group).

**Fig 6:**
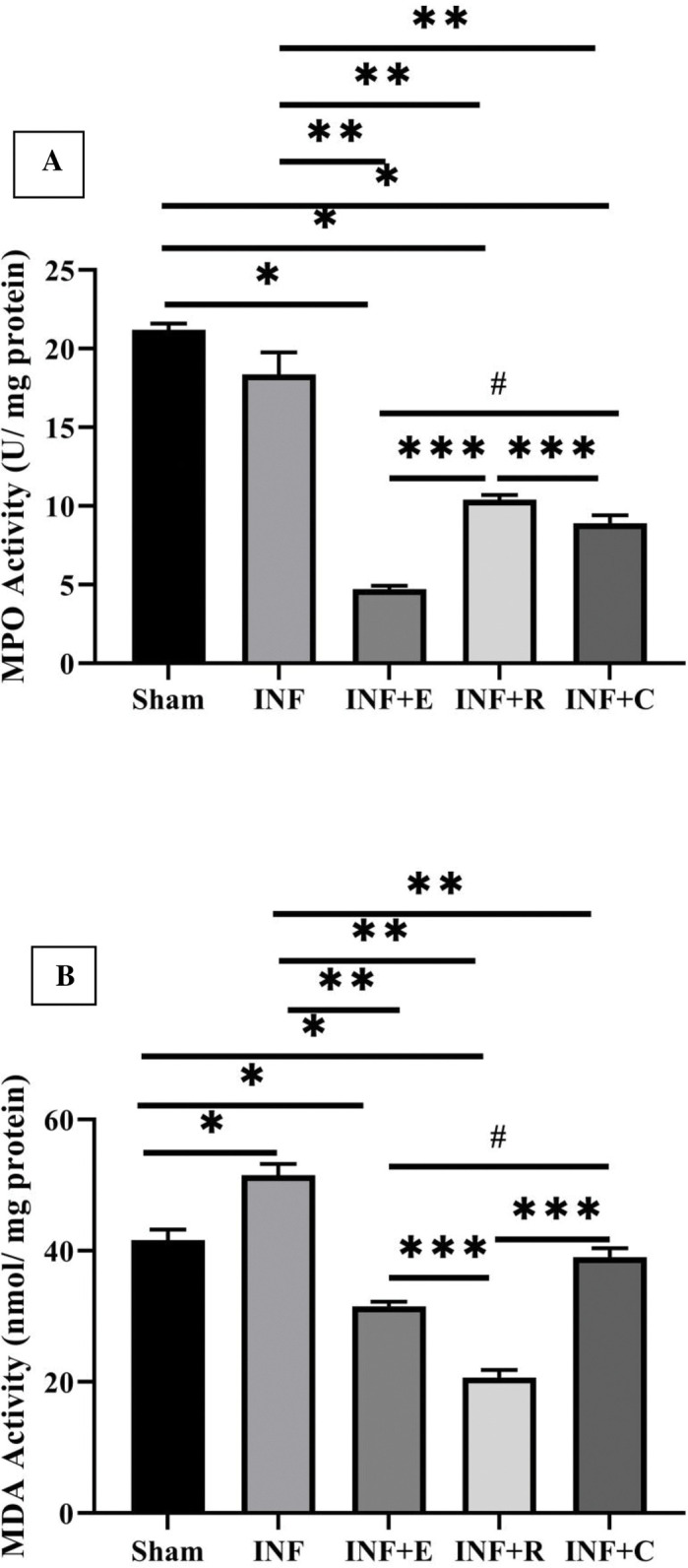
Effect of exercise preconditioning on the cardiac oxidant level. A) Activity of myeloperoxidase. B) Activity of malondialdehyde. Groups: The sedentary sham group, the INF (infarcted) control group, INF+ E (infarcted rats with endurance exercise intervention), INF+ R (infarcted rats with resistance exercise intervention), and the INF+ C (infarcted rats with concurrent exercise intervention. *P< 0.05, ***P< 0.001 & ****P< 0.0001 vs. INF or control group. Bars represent the means ± SEM (N = 5/group).

As shown in Figs 5 and 6, exercise intervention increased the activity of antioxidant enzymes and reduced the activity of oxidative enzymes significantly. There was a difference in Cardiac MPO among groups (χ2 = 22.5, p = 0.0001). All three exercise modes significantly decreased the MPO enzyme activity. The reduction of MPO in endurance training was more than in resistance and concurrent training groups, and cardiac MPO in concurrent groups was less than in the resistance group (p = 0.032).

Cardiac MDA was different between groups (χ^2^ = 22.14, p = 0.0001). Ischemia-reperfusion promoted cardiac MDA activity (p = 0.009). Regarding the enzyme activity of MDA, Kruskal-Wallis H and U Mann Whitney tests showed a reduced enzyme activity in all three training modes. The reduction of MDA activity in the resistance training group was more than in aerobic and concurrent training groups.

## 4. Discussion

The results of this paper showed that exercise preconditioning modifies the infarction size, factors associated with angiogenesis, and redox status. This study has directly compared the impact of three different methods of exercise preconditioning on the heart. The cardioprotective mechanisms and new findings of this study can be thus summarized as follows: 1) as a result of various training methods, there was a significant decrease in the infarction size following the regional ischemia-reperfusion; however, different exercise methods did not affect the reduction magnitude of the myocardial infarct size. 2) The aerobic and concurrent exercises significantly decrease circulating CK-MB_2_. 3) The endurance exercise increases the cardiac angiogenesis factors of VEGF, ANGP-1, and ANGP-2 and decreases the cardiac oxidant levels of MDA and MPO. 4) The resistance exercise increases the cardiac angiogenesis factor of VEGF and ANGP-2, decreases the cardiac oxidant levels (MDA, MPO), and increases the antioxidant activity of GPX. 5) Concurrent exercise increases the cardiac angiogenesis factor of VEGF and decreases the cardiac oxidant levels (MPO).

The cardioprotective effects of exercise training in cardiovascular disease in humans and animals are well established [39–42]. The reduction of the infarction size has been observed following a prior exercise routine in infarcted animals in various investigations [43–47]. McDonald and coworkers reported that the extent of exercise-related protection from ischemia-reperfusion injury depends on exercise modality [48], but the mode of exercise with the most protective effects against ischemia-reperfusion injury is unknown.

Barboza et al. revealed the beneficial effects of aerobic and dynamic resistance training against detrimental changes caused by myocardial infarction that lead to the reduction of inflammatory cytokine in the left ventricle [49].

This study investigates the effects of a combined training model, considering the distinctive protective mechanisms of endurance and resistance training on the heart, given that the impacts of preconditioning through these three exercise models have not been compared yet.

The concurrent training effect is an adaptation compromise, which appears to be most affected by the interference of the molecular pathways in the underlying adaptations from each type of training segment [50]. According to the results, concurrent training induces exercise preconditioning due to the protective mechanisms of both endurance and resistance training models.

Due to this study, all three kinds of training could attenuate the infarct size. These results, in turn, support the positive preconditioning effects of these three training models, which is of great importance because most sports use concurrent training.

The circulating biomarker that identifies cardiac damage is CK-MB_2_, which is highly regulated in cardiac tissue [51]. The highest CK-MB_2_ serum level is reached in 24 hours following an ischemic incident, dropping to the basal level in the next 72 hours [52]. In our study, High CK-MB_2_ levels indicate cardiac tissue damage. Unlike resistance training, reduced serum CK-MB_2_ levels in endurance and concurrent training demonstrate protective effects against cardiac damage. Regarding Feng et al. investigation, the CK-MB_2_ level decreased in rats inflicted with myocardial ischemia that underwent early aerobic exercise for three weeks compared to the sedentary infarcted animals [53]. In another study, Long-term exercise preconditioning decreased the CK-MB level significantly compared to those in the control group [54]. As other papers represent, our study also showed a decrease in the CK-MB_2_ index resulting from endurance and concurrent training. The reason that resistance training could not reduce the circulating CK-MB_2_ is probably due to the nature of resistance training, which leads to an increase in circulating CK-MB [55,56]. The elevation of circulating CK-MB_2_ despite infarction size reduction should be considered a marker of myocardial adaptation and not be interpreted as a heart injury [55].

As an angiogenesis growth factor, VEGF plays a crucial role in post-myocardial salvage by increasing capillary density. Wu et al. demonstrated that exercise elevates the VEGF levels in a time-dependent manner in infarcted mice. Concerning the study, exercise preconditioning before ischemic injury increased VEGF expression at all stages at mRNA and protein levels [57]. These results confirmed our experiments for all modes of exercise routines, indicating the upregulation of the VEGF expression. The expression of an angiogenic factor due to preconditioning has been shown to reduce ischemic injuries to the brain [58]. VEGF and angiopoietins are believed to be the principal vascular regulators, with ANGP-1 critical to the mutation of the newly formed vessels and ANGP-2 acting as its natural inhibitor [59,60]. These factors stabilize and remodel the vascular structure through endothelial cell survival, proliferation, migration, and tube formation [58]. Our study showed that exercise preconditioning has similar effects on angiogenic factors in the heart. However, training modes had different outcomes regarding these factors. VEGF increased in all exercise modes, but ANGP-1 protein increased significantly under endurance training, and ANGP-2 elevated in response to aerobic and resistance training. Considering these results, aerobic training is a more significant stimulus than resistance and concurrent training for the comprehensive development of angiogenesis after ischemia-reperfusion. In addition to angiogenesis, oxidative stress plays an essential role in ischemia-reperfusion injury and is one of the most crucial pathological mechanisms in ischemic injury [61]. A growing body of evidence indicates that oxidative stress causes myocardial damage during ischemia-reperfusion and subsequent remodeling through the activation of pathological pathways such as necroptosis, apoptosis, and inflammation [62]. The redox status is regulated via the production of free radicals and antioxidants. Our findings showed that resistance training affects both factors to reduce oxidative stress (elevation of antioxidant indices and lipid peroxidation reduction). On the other hand, endurance and concurrent modes moderate only one of the involved factors. Concurrent exercise promoted antioxidant indices, and endurance training reduced lipid peroxidation.

As a limitation of this study, the duration of reperfusion was short, and extended reperfusion periods (e.g., 24 hours) are needed to ensure proper detection of infarction. Chang et al. [63] demonstrated that the duration of reperfusion was an influential factor in the infarction size in normal rats, but the remarkable point in these findings was that in ligation for 30 minutes, the infarction size was 41.3 ± 2.1, 39.7 ± 0.7, 40.0 ± 1.2, 42.0 ± 1.3, and 42.0 ± 1.5 for 30, 120, 180, 270, and 360 minutes of reperfusion, respectively. Infarction size was very close between 30 and 360 minutes of reperfusion. A minimal 24h of reperfusion is required to provide solid results. In addition, VEGF increases newly formed vessels in a time-dependent manner. Previous studies showed that at least 72 hours are needed to complete the neovascularization process and neovascular sprouts [64]. Also, the fact that the weight of the heart slices is not considered in the infarction size and sizes of the area at risk is another limitation of this study.

## 5. Conclusion

In general, the cardioprotection of exercise training against ischemia-reperfusion injury depends on exercise modality. Cardioprotective effects of aerobic, resistance, and concurrent exercises vary based on their unique physiological mechanisms. Endurance training is more dependent on the angiogenesis process. Resistance training is more dependent on oxidative stress amelioration through antioxidant reinforcement and lipid peroxidation reduction. Furthermore, the preconditioning effect of concurrent exercise depends on both angiogenesis and oxidative stress amelioration.

## Supporting information

S1 File (XLS)

S2 File (XLS)

S3 File (XLS)

S4 File (XLS)
